# Revisiting the Discovery of the αβ TCR Complex and Its Co-Receptors

**DOI:** 10.3389/fimmu.2014.00583

**Published:** 2014-11-21

**Authors:** Ellis L. Reinherz

**Affiliations:** ^1^Laboratory of Immunobiology, Department of Medical Oncology, Dana-Farber Cancer Institute, Boston, MA, USA

**Keywords:** adaptive immunity, thymic development, co-receptors, TCR, CD3

This is an opinion article based on the paper “Clonotypic structures involved in antigen-specific human T cell function. Relationship to the T3 molecular complex”, by Meuer S. C., Fitzgerald K. A., Hussey R. E., Hodgdon J. C., Schlossman S. F., and Reinherz E. L. (1).

Life and science do not necessarily follow a straightforward path. So, it is not surprising, in retrospect, that my decision around Christmas of 1977 to terminate my clinical hematology fellowship in favor of a laboratory research associate position at Dana-Farber Cancer Institute working on lymphoid malignancies would result in progress in basic immunology. Remarkably, however, the outcome of the research yielded new insights into thymic development, mature T-cell heterogeneity, and the molecular basis for cognate recognition by T lymphocytes.

At the time, my decision was motivated by a clinical observation and desire to understand its basis. Namely, if a physician treated 100 children with acute lymphoblastic leukemia (ALL) using the same multi-agent chemotherapy, 80 of them would go into remission and 20 of them would die. I asked myself if that latter outcome was 100% of 20% vs. 20% of 100%. I thought that the answer might be the former but sought the opinion of a clinical mentor. He looked at me as if I had three heads and simply commenting that those who die have “poor protoplasm.” This abject ignorance was so appalling and annoying to me at the same time that I quit on the spot. Since I had bluntly told him what I thought of his response, I likely would have been fired if I had not voluntarily chosen to move on down the road quite literally, as it were. While I had no explicit experimental plans, it occurred to me that antibodies raised against these tumor cells might be capable of distinguishing subpopulation heterogeneity, should it exist. After all, in 1977, people were not distinguishing red blood cells from different individuals by holding them up to the light. In the 1920s, a man named Landsteiner sorted out differences among RBCs that looked alike through development of red blood cell typing technology (2).

In any event, I moved to Dana-Farber Cancer Institute to work with Stuart Schlossman, who was Chief of the Division of Tumor Immunology, which included his own laboratory, that of Harvey Cantor, and several others. Stu enthusiastically greeted me at the time. He was appreciative of my query since he, himself, was trained as a hematologist and attempting to dissect normal lymphoid heterogeneity. His laboratory already was generating rabbit antisera against various types of hematopoietic cells. He told me that he was just starting the production of monoclonal antibodies (mAbs) using the Kohler and Milstein method (3) and that I might get involved. I retrieved human thymuses from children and neonates undergoing open heart surgery for congenital cardiac abnormalities, peripheral T-cells from normal volunteers isolated by sheep erythrocyte rosetting (a way of fractionating human T-cells), and a host of leukemic populations from my earlier patients. We used human cells to immunize mice in an effort to stimulate antibody production. The combination of the species differences as well as FACS-based screening of B-cell hybridoma supernatants *in lieu* of radioimunoassays employed by most other groups permitted us to quickly identify antibody targets, even when not expressed at high levels on cells or restricted to a subpopulation of those cells being interrogated. As a consequence, it was rather simple to derive mAbs against targets on subpopulations of mature and immature T lineage cells.

In 1979, we first identified the CD4 molecule, which we found to be expressed on two-thirds of peripheral mature T lymphocytes with helper activity (4–6) and then CD8 molecules expressed on the reciprocal subset of T-cells, which manifest most of the cytotoxic activity (7). In contrast, within the thymus itself, we originally described the major population of thymocytes co-expressing CD4 and CD8, which we termed double positive (DP) as precursors of mature thymocytes (8). In addition, we observed a small subset of CD4^−^CD8^−^ (double negative, DN) thymocytes. The vast majority of ALLs refractory to chemotherapy (those 20% above) was derived from the DN thymocytes (8). Susceptibility of this DN thymocyte population to activating mutations in NOTCH and aberrations of competitive niches created during early development are currently evolving as explanations of thymocyte susceptibility [(9) and references therein]. The fact is even without detailed molecular understanding of these immature ALLs and more mature T lineage malignancies like acute lymphoblastic lymphoma and Sezary syndrome, it was obvious that such tumors represented frozen states of normal T lineage development. The notion that thymocyte development progressed from DN to DP to SP (CD4^+^CD8^−^ or CD4^−^CD8^+^) derived from our studies. Acceptance of this idea had to wait more than 4 years for the mouse immunologists to create the anti-murine CD4 mAb equivalent, L3T4 (10). The use of these mAbs revolutionized mature T-cell subset characterization in human beings offering CD4/CD8 clinical ratios, absolute CD4 counts, and the like. Comparative analysis done with Lorenzo Loretta and Max Cooper using other then currently accepted methods revealed that the new approach to define T lymphoid heterogeneity with mAbs was superlative to those existing technologies (11).

In 1980, we first observed that the anti-CD3 mAb could block antigen-specific human T-cell proliferation to both soluble antigens and alloantigens, as well as inhibit generation of cytotoxic T-cells (12). Of note, CD3 was expressed at latter stages of thymic development but maintained on all mature peripheral T-cells. Moreover, antigen recognition by human T lymphocytes was linked to surface expression of the CD3 molecular complex. When human T-cell clones were incubated with anti-CD3 mAb at 37°C, there was a rapid and selective loss of CD3 expression and concomitant antigen unresponsiveness (13). The latter was not a generalized cellular inhibition since IL-2 responsiveness remained intact. Removal of anti-CD3 from cell culture mAb was followed by restoration of T-cell surface CD3 expression and, in parallel, return of antigen responsiveness. These data set the stage for Ortho/Johnson & Johnson Pharmaceuticals to develop OKT3 as a human immunosuppressive therapeutic that was tested in treatment of allograft transplant rejection and became the very first FDA approved mAb in 1985 (14, 15).

Because research productivity went well, I was promoted to Assistant Professor in the Medicine Department at Harvard Medical School in 1980. Baruj Benacerraf had both taken over the helm of DFCI that year as its president and won the Nobel Prize in Physiology or Medicine in 1980 with Jean Dausset and George Snell for “their discoveries concerning genetically determined structures on the cell surface that regulate immunological reaction,” i.e., MHC. As a newly minted faculty member, Baruj summoned me to his office so that I could describe to him the plans for my fledgling research operation. I told Dr. Benacerraf that all of my efforts were going to focus on defining T-cell antigen recognition, including identification of the T-cell receptor. He told me that this goal was ambitious, bold but probably ill advised. There were too many established laboratories working on this central immunological problem. “What makes you think it’s likely that you will succeed over them?” he queried. I responded: “I’m looking for the receptor on the surface of a T cell, not in culture supernatants as the others are doing.” He shrugged, wished me luck, and so the next phase began. In the several years that followed when success was achieved, it should be noted that Baruj was laudatory and glad I followed my scientific conviction.

In 1982, by exploiting T-cell cloning techniques, first described by Smith and colleagues (16), alloreactive CTL could be derived from both human CD4 and CD8 subsets. Strikingly, CD4 T-cells recognized MHC class II products, whereas CD8 T-cells recognized MHC class I products. These cells could be blocked by appropriate anti-MHC I/II or anti-CD4 or anti-CD8 antibodies (17–22). Given that anti-CD3, anti-CD4, and anti-CD8 mAbs all blocked CTL activity, we wondered whether the surface molecules identified by these mAbs detected recognition elements or, alternatively, components of the lytic machinery. Lectin approximation studies excluded the lytic machinery option since even in the presence of the blocking antibodies, lectin restored CTL function, although pointedly with loss of target specificity.

The fact that biochemical analysis [see, for example, in Ref. (23, 24)] failed to identify differences in peptide maps or electrophoretic mobility of these molecules on human T-cell clones of differing specificities argued that they were invariant structures incapable of conferring antigen and MHC specificities *per se*. Because each cloned T lymphocyte recognizes antigen in a precise fashion, one could not account for its unique specificity on the basis of monomorphic portions of CD3, CD4, or CD8 (1). I reasoned that there had to exist discriminative surface structures on individual clones, which we refer to as clonotypes or idiotypes. mAbs to such idiotypic structures (T idiotypic = Ti) were produced next by immunizing mice with CTL clones, screening the resulting antibodies on the immunizing CTL and then selecting those which lacked reactivity with additional clones of different antigen specificities from the same donor (1, 25, 26). Such antibodies were unique in that they inhibited cell-mediated killing and antigen-specific proliferation of the individual immunizing clone without affecting the function of other autologous clones. Moreover, like anti-CD3 mAbs, the anti-clonotypic antibodies enhanced IL-2 responsiveness and induced modulation of the Ti structure with CD3. Data showed that the Ti clonotype was closely associated with CD3 in the membrane of human T-cells. Immunoprecipitation and competitive binding analysis revealed that the anti-clonotypes defined a disulfide-linked heterodimer with α and β subunits of approximately 49 and 43 kD, respectively. The heterodimeric clonotype was not physically associated with CD4 or CD8 but was in non-covalent association with the invariant CD3 molecules as a complex first evidenced by our analyses (1, 23, 25, 26).

From the above data collectively, I proposed with my colleagues a working model of T-cell cognate recognition (27) in which the antigen binding structure comprised a clonally unique αβ heterodimeric Ti moiety in complex with CD3. The associative recognition element is either CD4 or CD8 depending on the subset derivation of the individual T lymphocyte. In this model, CD4 and CD8 accessory (“co-receptor”) glycoproteins bind to constant regions of class II or class I MHC, respectively, which are separate from the CD3-linked clonotype. The TCR complex, on the other hand, was defined as a CD3-associated Ti αβ heterodimer working in concert with CD4 and CD8 to mediate MHC-restricted antigen recognition. This view implied that there was a bidentite interaction of the TCR complex and co-receptor with the same peptide/MHC. This proposal has been codified in structural studies over the last 30 years [for review, see Ref. (28)]. Confidence that Ti was the αβ TCR heterodimer encoding both peptide and MHC specificities came from (1) the unique ability of anti-clonotypic mAbs coupled to Sepharose beads to trigger T-cell clones, replacing requirements for cognate antigen plus MHC (29); (2) biochemical evidence for peptide variability within α and β subunits of Ti (30, 31), implying existence of constant and variable regions as found in Ig heavy and light chains; (3) αβ purification and amino acid sequencing showing Ig homologies for each subunit (32, 33); (4) the putative TCR triggering resulted in T-cell proliferation via an IL-2-dependent mechanism not observed by crosslinking other T-cell structures (34); and (5) direct evidence for the existence of nominal antigen binding sites on Ti αβ heterodimers of MHC-restricted T-cell clones specific for fluorescein-5-isothiocyanate (35).

The work carried out over a period of these several years was extraordinarily exciting, converting concepts into explicit molecular identities, beginning to explain the complexities of T-cell recognition, providing reagents for clinical efforts and fodder for considerable future structural and molecular studies. These TCR efforts required a spirited collection of colleagues including Stefan Meuer who developed T-cell clones and performed many functional studies with Rebecca Hussey who made the various mAbs used in the majority of these studies, and Oreste Acuto who led the biochemical charge on the TCR complex and TCR αβ heterodimer purification and amino acid sequencing with Marina Fabbi. Bob Siliciano then showed that the TCR αβ heterodimer actually bound ligand.

Our efforts on TCR biology and its identification were complemented very soon by studies in the mouse by Pippa Marrack and John Kappler using T-cell hybridomas. More explicitly, we published in JEM in February 1983 on the first anti-clonotypic mAb (1), while they published in the same journal in April 1983 (36). Comparisons of TCR αβ heterodimer clonotypes were published by us in Nature in June 1983 and in PNAS in July 1983 (23, 25) whereas their comparison appeared in Cell in October 1983 (37). We published on the ability of ant-clonotypic mAbs to replace the requirement of peptide and MHC in T-cell activation in September 1983, whereas our competitors showed that anti-clonotypic antibody binding to T-cell hybridomas predicted antigen and MHC specificity in November 1983 (38). Furthermore, their biochemical data matched well with that of the human being and an independently identified disulfide-linked T-cell tumor-specific antigen identified with a mAb produced by Allison et al. (39). The human being was a particularly informative and tractable species choice since we had created reagents that defined the TCR αβ heterodimer, CD3 components, and CD4 and CD8 co-receptors. The majority of reagents defining these receptors was lacking in the mouse at the time. Biochemical detail by Terhorst and Klausner further refined the nature of the CD3 components of the TCR complex (40, 41). The objective impact of the three Reinherz, Marrack, and Allison group efforts from the 1980 to 2000 time period is evident from ISI citations (20,000 vs. 8,000 vs. 900, respectively). In turn, molecular cloning of the TCR subunits using a subtractive hybridization method began (42–45). These studies identified TCRβ as shown by our subsequent N-terminal amino acid sequencing analysis (32). More than 30 years later, however, we are still in the process of detailing structure and function of the TCR complex and its co-receptors. The trove of information herein will lead to important therapeutic inventions for treatment of autoimmune and immunodeficiency diseases to be fully realized in the coming years.

## Conflict of Interest Statement

The author declares that the research was conducted in the absence of any commercial or financial relationships that could be construed as a potential conflict of interest.
